# The Combined Inhibition of SREBP and mTORC1 Signaling Synergistically Inhibits B‐Cell Lymphoma

**DOI:** 10.1002/cam4.70342

**Published:** 2024-11-05

**Authors:** Zhenhan Zhu, Wenxia Jiang, Jiehao Zhou, Alexander Robert Maldeney, Jingru Liang, Jing Yang, Wei Luo

**Affiliations:** ^1^ Department of Microbiology and Immunology Indiana University School of Medicine Indianapolis Indiana USA; ^2^ Department of Laboratory Medicine and Pathology Mayo Clinic Arizona Phoenix Arizona USA; ^3^ Department of Biochemistry and Molecular Biology Indiana University School of Medicine Indianapolis Indiana USA; ^4^ Indiana University Simon Comprehensive Cancer Center Indiana University School of Medicine Indianapolis Indiana USA; ^5^ Indiana University Cooperative Center of Excellence in Hematology (CCEH) Indiana University School of Medicine Indianapolis Indiana USA

## Abstract

**Background:**

The sterol regulatory element‐binding protein (SREBP) pathway is essential for maintaining sterol homeostasis during B cell activation and germinal center B cell proliferation. However, its potential as a therapeutic target to treat B‐cell lymphoma remains unclear.

**Methods:**

We examined SREBP protein expression in human B‐cell lymphoma samples using immunohistochemistry. Additionally, we conducted in vitro studies using SREBP signaling inhibitors in combination with rapamycin to assess their effects on cell proliferation and lipid metabolism in B‐cell lymphoma cells.

**Results:**

Our analysis revealed high levels of SREBP2 protein expression in human B‐cell lymphoma samples. Inhibiting SREBP signaling or its downstream target HMG‐CoA reductase (HMGCR) with Fatostatin or Simvastatin effectively suppressed B‐cell lymphoma cell proliferation. However, B‐cell lymphoma cells responded to statin treatment by activating the mTORC1‐pS6 pathway, suggesting a compensatory mechanism to overcome statin‐induced cell cycle arrest. Combining low‐dose statin treatment with the mTOR inhibitor rapamycin produced a synergistic effect, significantly inhibiting B‐cell lymphoma proliferation, cell cycle progression, and lipid raft formation.

**Conclusions:**

These results highlight the potential of a combined therapeutic approach targeting both SREBP and mTORC1 as a novel strategy for treating B‐cell lymphoma.

## 1 Introduction

The sterol regulatory element binding proteins (SREBP) are controlled by SREBP cleavage activating protein (SCAP), a sensor of intracellular cholesterol levels [1]. The depletion of cholesterol activates SCAP, which escorts SREBP from the endoplasmic reticulum (ER) to the Golgi, triggering their activation and nuclear translocation [1, 2]. SREBP proteins target a set of genes specialized in lipid biosynthesis. Downstream of SREBP, cholesterol biosynthesis involves a rate‐limiting enzyme HMG‐CoA reductase (HMGCR) [3, 4]. Altered cholesterol and lipid metabolism are associated with various cancers [5, 6, 7]. How to target lipid metabolism to effectively treat cancer remains a critical question.

In our previous studies, we found that SREBP signaling is dispensable for B‐cell development and maintenance at steady state. However, the depletion of SREBP signaling in B cells lead to an inability to effectively form germinal centers (GC) or bone marrow plasma cells in response to immunization or viral infection [8]. Mechanistically, SREBP signaling is essential for maintaining lipid homeostasis and metabolic reprogramming during B‐cell activation and proliferation. Disrupting SREBP signaling specifically in GC B cells inhibited cell cycle progression and reduced lipid raft content [8]. B‐cell lymphomas are often originated from the GC. We found that SREBP2 is highly expressed in human B‐cell lymphoma samples. However, the role of SREBP signaling in regulating the pathogenesis of B‐cell lymphoma cells remains unclear.

To investigate this, we utilized statin drugs to inhibit the SREBP‐HMGCR axis and conducted in vitro studies on representative germinal center B‐cell (GCB) and activated B‐cell (ABC) subtypes of diffuse large B‐cell lymphoma (DLBCL) cell lines. Our findings reveal that inhibiting SREBP with fatostatin or inhibiting HMGCR with simvastatin at a high dose can impede cell proliferation of B‐cell lymphoma. However, in response to SREBP inhibition, B‐cell lymphoma cells activate mTORC1 signaling, as demonstrated by the substantial phosphorylation of S6, indicating a potential pathway for overcoming the inhibitory effects of statin treatment. Consequently, the combined application of statin with an mTOR inhibitor rapamycin, even at a low dose, can strongly suppress B‐cell lymphoma proliferation. These studies present a promising alternative treatment for B‐cell lymphoma by combining two clinically approved drugs.

## 2 Results

### 2.1 SREBP Signaling Is Altered in Human DLBCL

**FIGURE 1:**
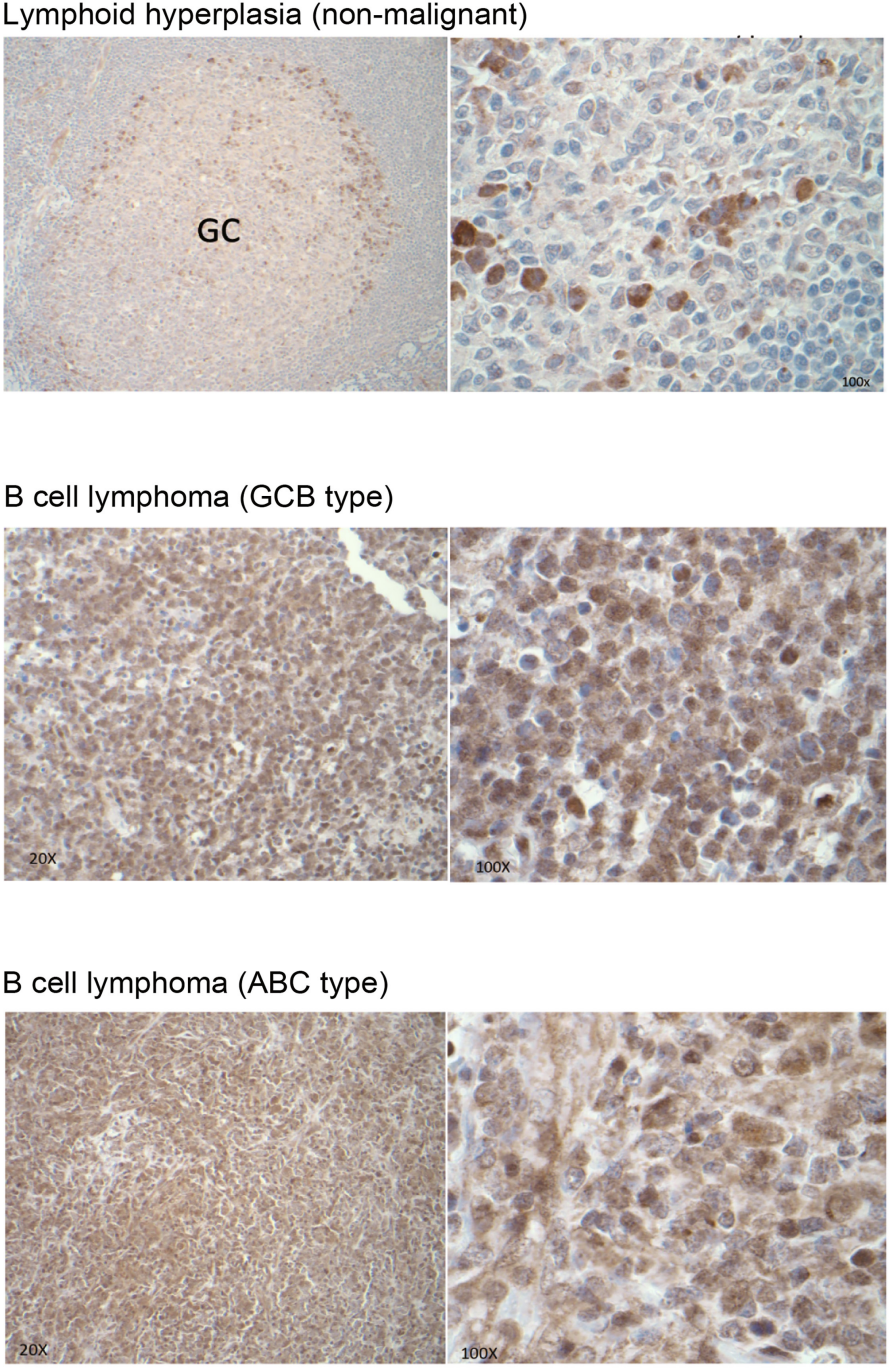
SREBP2 IHC staining comparing human non‐malignant lymphoid tissue and B‐cell lymphoma samples.

The SREBP pathway is crucial for lipid biosynthesis, with SREBP2 primarily involved in cellular cholesterol generation. To investigate the role of SREBP2 in human B‐cell lymphoma cells, we first conducted immunohistochemistry (IHC) staining to assess the levels of SREBP2 expression in human B‐cell lymphoma samples. Our findings revealed that, in comparison to non‐malignant lymphoid tissue, SREBP2 protein expression was highly elevated in both GCB‐like and ABC‐like DLBCL samples (Figure 1).

### 2.2 SREBP‐HMGCR Axis Is Essential for Cell Cycle Progression of B‐Cell Lymphoma Cells

**FIGURE 2:**
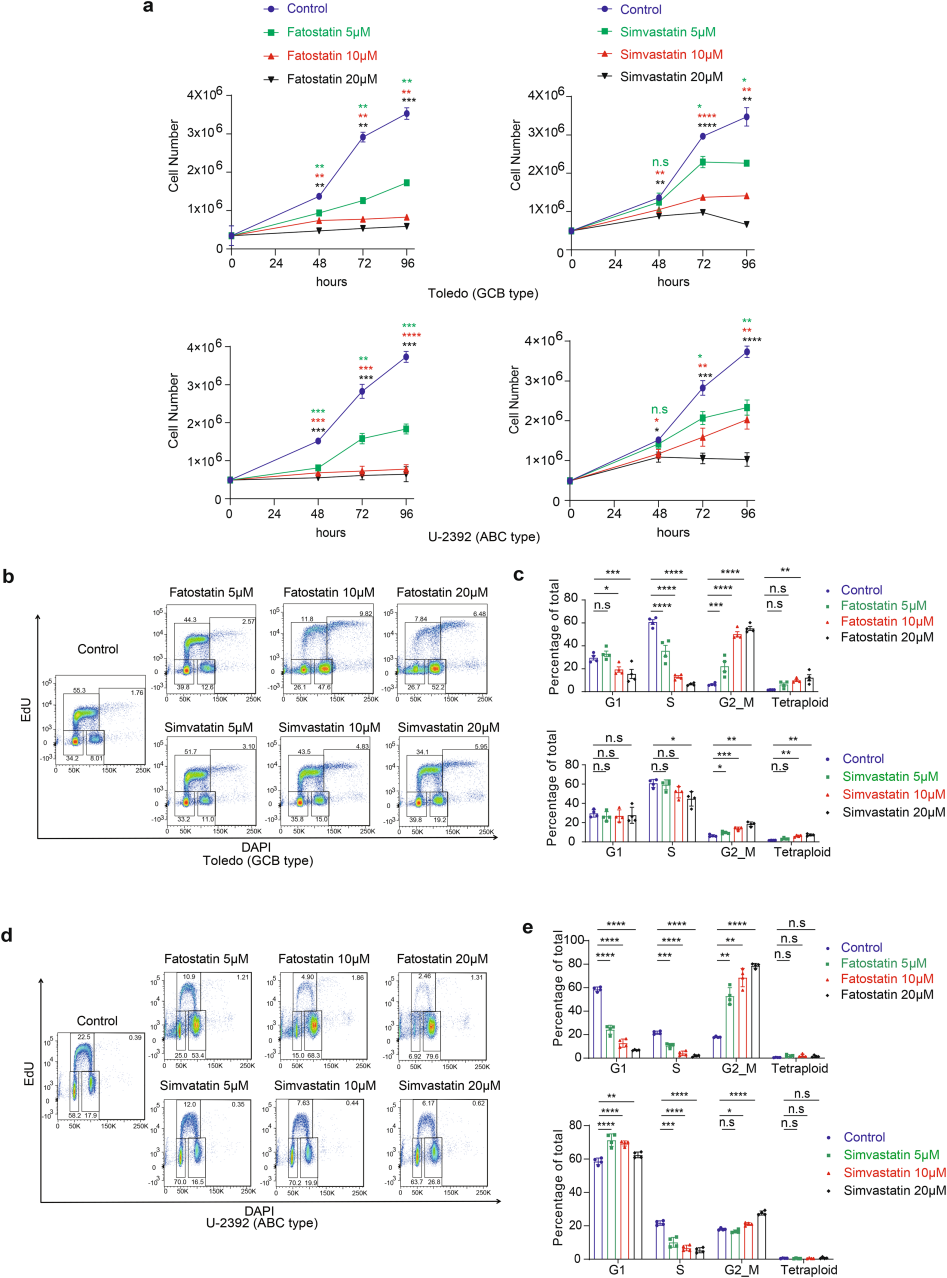
SREBP‐HMGCR inhibition on B‐cell lymphoma cell proliferation and cell cycle progression. (a) Cell proliferation curves under different concentrations of fatostatin (5 μM, 10 μM, 20 μM) and simvastatin (5 μM, 10 μM, 20 μM) treatments. (b–e) Toledo and U‐2392 cells were treated with fatostatin (5 μM, 10 μM, 20 μM) or simvastatin (5 μM, 10 μM, 20 μM) for 48 h. Cell cycle phases (G1, S and G2/M) were analyzed by FACS with EdU and DAPI. (b) and (d) show representative FACS plots; (c) and (e) show statistical analysis. *p* values were determined by two‐way ANOVA followed by Dunnett's multiple comparisons test (a) or one‐way ANOVA followed by Tukey's multiple comparisons test (c, e). *p ≤ 0.05; **p ≤ 0.01; ***p ≤ 0.001; ****p ≤ 0.0001; n.s, not significant (*p* > 0.05).

To investigate whether the altered SREBP signaling plays a role in the pathogenesis of B‐cell lymphoma, we inhibited this pathway using statin drugs to explore their potential therapeutic effects on B‐cell lymphomas. Fatostatin inhibits the activation of SREBP proteins [9], while simvastatin more specifically inactivates HMGCR [10], a downstream target of SREBP proteins. Toledo (GCB subtype) and U‐2392 (ABC subtype) B‐cell lymphoma cell lines were treated with different concentrations (5 μM, 10 μM, 20 μM) of fatostatin and simvastatin for 4 days. We observed that inhibiting either upstream or downstream of this sterol synthesis pathway can suppress the proliferation of B‐cell lymphoma cell lines in a dose‐dependent manner (Figure 2a).

To further investigate the impact of statin drugs on cell cycle progression of B‐cell lymphoma. Toledo and U‐2392 cell lines were treated with different concentrations fatostatin and simvastatin for 48 h, followed by cell cycle analysis using EdU and DAPI staining. As shown in Figure 2b–e, inhibiting SREBP signaling with these statins, particularly at high doses, effectively reduced the frequencies of cells in the S phase. Additionally, lymphoma cells treated with statins exhibited an accumulation in the G2‐M phase, suggesting that the inhibition of SREBP signaling can impede cell growth and/or mitosis. Notably, for Toledo, a significant increase in tetraploid cells was observed with statin treatment (Figure 2b,c), indicating that the cells were unable to undergo mitosis [11]. These findings strongly support that sterol synthesis by SREBP‐HMGCR axis is a checkpoint regulator for the cell cycle progression of B‐cell lymphoma cells.

### 2.3 Activation of mTORC1 Signaling in B‐Cell Lymphoma Treated With Statins

**FIGURE 3:**
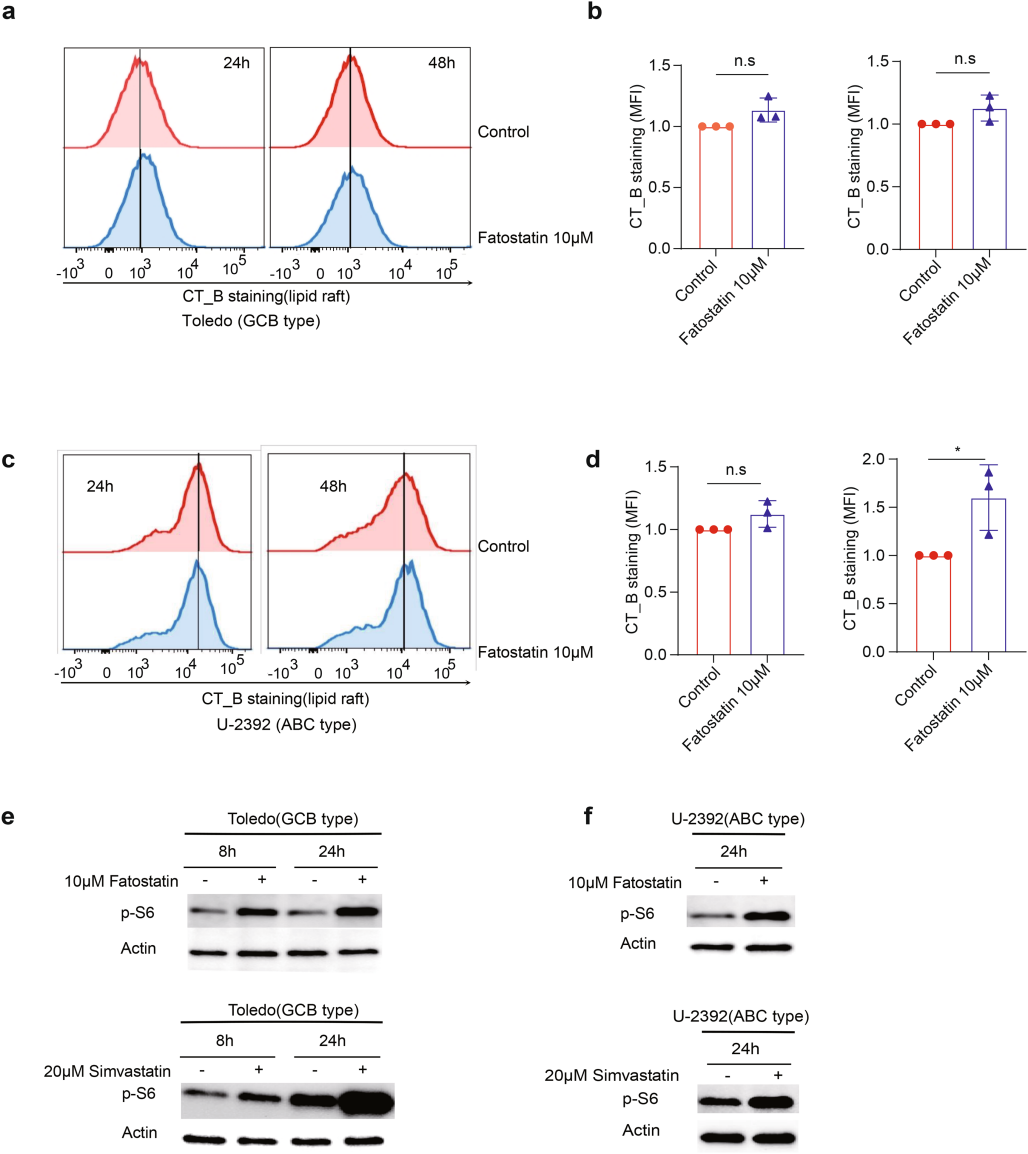
The impact of SREBP signaling inhibition on lipid rafts and mTOCR1 signaling in B‐cell lymphoma cells. (a–d) Toledo and U‐2392 cells were treated with fatostatin (10 μM) for 48 h. Lipid rafts were analyzed by FACS with CT‐B staining. Shown are representative histograms of CT‐B staining and statistical analysis from three independent experiments. Median fluorescence intensity (MFI) values were normalized to control samples (set as 1). Statistical significance was determined by two‐tailed unpaired t‐test. *p ≤ 0.05; n.s, not significant (*p* > 0.05). (e, f) Toledo and U‐2392 cells were treated with fatostatin (10 μM) or simvastatin (20 μM) for indicated time points. Immunoblotting was used to compare p‐S6 expression between treated and untreated cells.

In our earlier study, we demonstrated that SREBP signaling is crucial for maintaining lipid raft content in non‐malignant activated B cells and in GC B cells [8]. Building on this observation, we investigated whether inhibiting SREBP‐HMGCR axis through statins could similarly disrupt lipid rafts in B‐cell lymphoma cells. To assess this, we employed cholera toxin subunit B (CT‐B) staining, a marker for lipid rafts [12]. Surprisingly, our findings revealed that statin treatments did not decrease lipid raft content in B‐cell lymphoma cells (Figure 3a–d). For U2392 cell line, 48‐h fatostatin treatment even slightly increased lipid raft content (Figure 3c,d). The data suggest that at this dosage, fatostatin is unable to fully inhibit sterol biogenesis.

To explain this unexpected result, we hypothesized that B‐cell lymphoma cells may activate alternative lipogenic pathways when SREBP signaling is inhibited, which may compromise the therapeutic effects from statin treatments. Given that mTORC1 signaling is known to play a role in lipid biosynthesis [13], we conducted western blotting to assess S6 phosphorylation, a readout for mTORC1 activation [14]. We found that both fatostatin and simvastatin treatments led to increased p‐S6 levels in both B‐cell lymphoma cell lines (Figure 3e,f). These data suggest that inhibiting SREBP signaling may induce alternative lipogenic pathways mediated by mTORC1 signaling, potentially a feedback mechanism used by cancer cells to compensate for the loss of SREBP signaling.

### 2.4 Combined Use of Low‐Dose Statins and mTORC1 Inhibitor Rapamycin Synergistically Inhibits B‐Cell Lymphoma Proliferation

Building on the observed feedback activation of mTORC1 signaling in B‐cell lymphoma treated with statins, we hypothesized that inhibiting mTORC1 might enhance the inhibitory effects of statins in B‐cell lymphoma. To test this hypothesis, we treated Toledo and U‐2392 B‐cell lymphoma cells with low doses of fatostatin or simvastatin, either alone or in combination with 10 nM mTORC1 inhibitor rapamycin.

**FIGURE 4:**
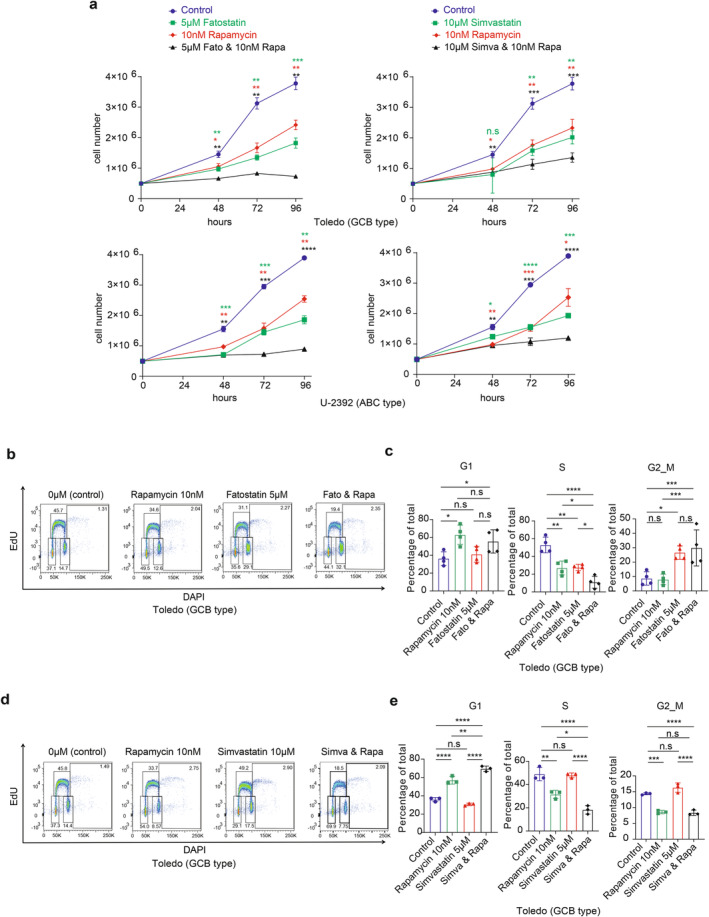
Synergistic inhibition of B‐cell lymphoma cells with combined SREBP and mTORC1 inhibition. (a) Cell proliferation curves under fatostatin (5 μM) or simvastatin (10 μM) treatment alone or in combination with rapamycin (10 nM) treatment. (b–e) Toledo cells were treated with fatostatin (5 μM) or simvastatin (10 μM) alone or in combination with rapamycin (10 nM) for 24 h. Then cell cycle (G1, S and G2/M) analyses were performed using EdU and DAPI staining. Representative FACS plots and statistical analysis of 3–4 independent experiments were shown. *p* values were determined by two‐way ANOVA followed by Dunnett's multiple comparisons test (a) or one‐way ANOVA followed by Tukey's multiple comparisons test (c, e). *p ≤ 0.05; **p ≤ 0.01; ***p ≤ 0.001; ****p ≤ 0.0001; n.s, not significant (*p* > 0.05).

As illustrated in Figure 4a, combined application of low doses of fatostatin (5 μM) or simvastatin (10 μM) with rapamycin (10 nM) synergistically inhibited B‐cell lymphoma cell proliferation. To further clarify the effects of these treatments on the cell cycle progression, we conducted EdU and DAPI staining. The findings demonstrated that the combined treatment using statin and rapamycin synergistically decreased the frequency of cells in the S phase (Figure 4b–e).

### 2.5 The Combinat Treatment of Statins and Rapamycin Result in a Reduction of Lipid Raft Content

**FIGURE 5:**
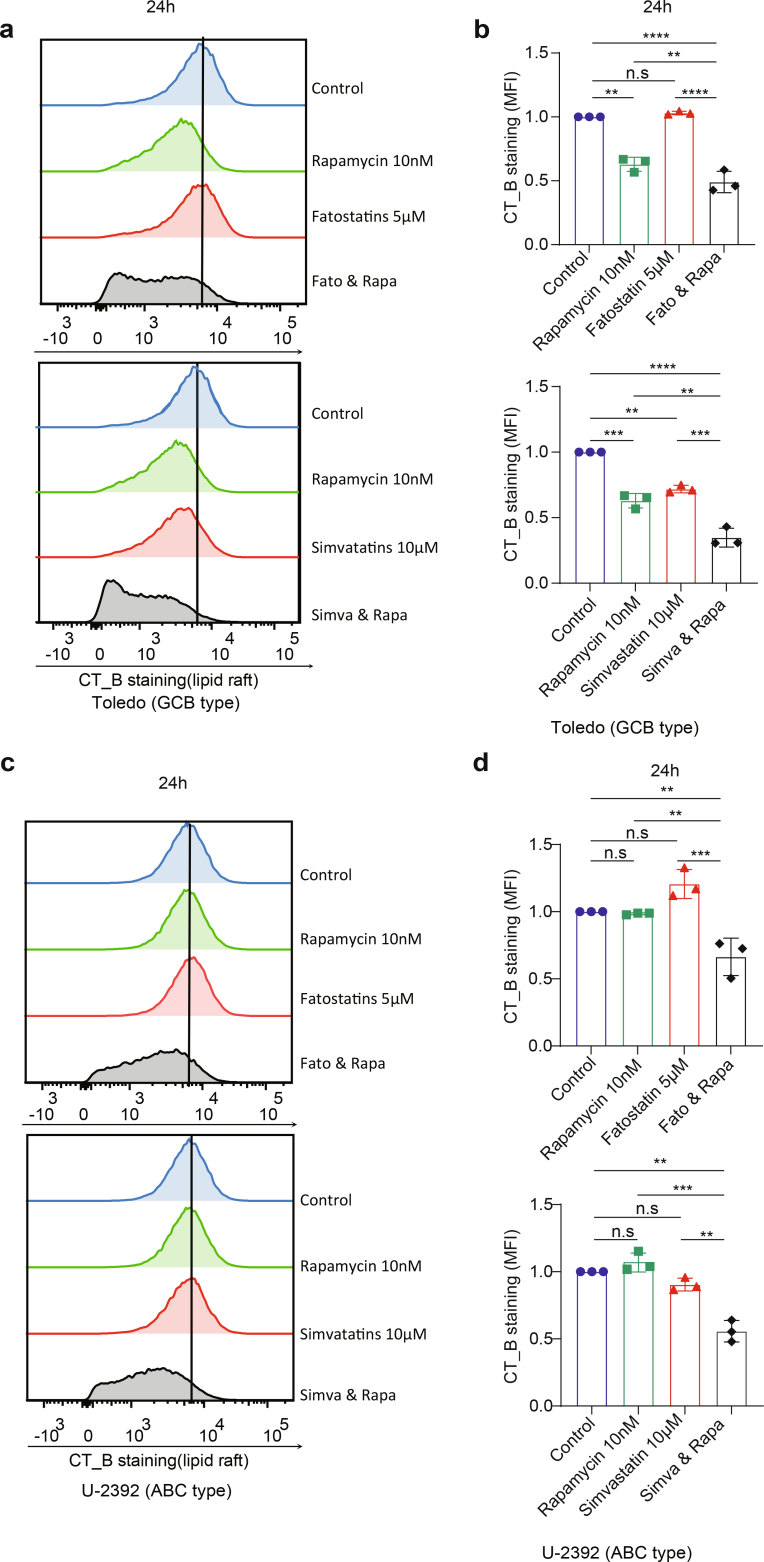
Combined SREBP and mTORC1 signaling inhibition reduces lipid raft content in B‐cell lymphoma cells. (a–d) Toledo and U‐2392 cells were treated with fatostatin (5 μM) or simvastatin (10 μM) alone or in combination with rapamycin (10 nM) for 24 h. Lipid rafts were analyzed by FACS using CT‐B staining. Shown are representative histograms of CT‐B staining and statistical analysis of three independent experiments. Median fluorescence intensity (MFI) values were normalized to control samples (given a value of 1). Statistical significance was determined by one‐way ANOVA followed by Tukey's multiple comparisons test. **p ≤ 0.01; ***p ≤ 0.001; ****p ≤ 0.0001; n.s, not significant (*p* > 0.05).

Considering these intriguing results, we investigated the formation of lipid rafts in response to combined treatments as compared to statin or rapamycin alone. As illustrated in Figure 5a–d, combined treatments of statin and rapamycin substantially reduced lipid raft content in B‐cell lymphoma cells. These findings imply that the dual inhibition of mTORC1 and SREBP signaling can highly impact lipogenesis, providing additional insight into the synergistic inhibitory effects on B‐cell lymphoma.

## 3 Discussion

Statin drugs have been repurposed to treat other cancer types in previous studies. However, therapeutic effects were only evident with high dose statin treatments (80 mg/day) [15, 16, 17]. Considering the side effects of statin drugs [18], the requirement of high dose for sufficient efficacy significantly limits the therapeutic applications of statins in cancer treatments.

In this study, we identified that the proliferation of B‐cell lymphomas is dependent on SREBP signaling. This aligns with numerous clinical studies indicating the reduced risk of B‐cell derived lymphomas, including DLBCL and plasma cell lymphoma, with statin use [17, 19, 20, 21]. Although statin drugs can suppress the proliferation of B‐cell lymphoma by inhibiting SREBP signaling, their optimal efficacy requires high doses, restricting therapeutic applicability. Using lipid raft content as a maker for lipid homeostasis, our research reveals that statin treatment by themselves may induce feedback regulation to maintain lipid homeostasis through a possible alternative pathway controlled by mTORC1 signaling. To validate this mechanism, we simultaneously inhibited of both SREBP and mTORC1 signaling, which resulted in a more potent suppression of lymphoma cell proliferation. These findings propose a feasible strategy for treating B‐cell lymphoma.

## 4 Methods

### 4.1 IHC Staining

Human lymphoma samples and non‐malignant hyperplasia lymphoid samples were sectioned and stained with SREBP2 antibody (ThermoFisher Cat #PA1‐338) for microscopy analysis.

### 4.2 B Cell Lymphoma Cells and Treatments

Toledo and U‐2392 cell lines were provided by Dr. Mark Sholmchik from University of Pittsburgh as gifts. Cells maintained at 37°C with 5% CO_2_ in culture medium (RPMI 1640 medium supplemented with 10% fetal bovine serum (FBS), penicillin/streptomycin, glutamine and 50 μM 2‐mercaptoethanol). Cells were used for experiments when reaching a density of 2 × 10^6^ cells per mL, with a viability greater than 95%. Cells (1 × 10^6^ cells mL^−1^) followed by the replacement of the medium with fresh culture medium. Fatostatin (Cat: B5599, ApexBio), simvastatin (567022, EDM Millipore Corp), and rapamycin (R64500, RPI) were dissolved in dimethyl sulfoxide (DMSO) to prepare stock solutions.

Lymphoma cells were plated at 5 × 10^5^ cells in 1 mL in 12‐well plates. Then, cells were treated with 5 μM, 10 μM, 20 μM of fatostatin or simvastatin for 8 to 96 h. For some experiments, cells were treated with rapamycin (10 nM) or rapamycin combined with statins. Control samples were treated with the same volumes of DMSO. For all treatments, DMSO concentration were ≤ 0.1%.

### 4.3 Immunoblotting Analysis

Whole‐cell lysates were prepared by direct lysing and boiling samples in Laemmli buffer supplemented with 2‐mercaptoethanol. The samples were subjected to immunoblotting and membranes were incubated with one of the following antibodies from Cell Signaling Technology: Actin (clone D18C11; 8456S), phospho‐S6 (Ser235/236) (clone D57.2.2E; 4858S). The signals on the membranes were captured using a Bio‐Rad ChemiDoc Imager and quantitated using Image Lab Software V6.1 (Bio‐Rad). Loading was normalized by blotting for actin.

### 4.4 Flow Cytometry

Cells were plated at 5 × 10^5^ cells per well in 12‐well plates and stimulated with EdU (10 μM) for 1‐h. Cells were stained for viability with Ghost Dye Violet 510 (Cytek Cat: SKU 13‐0870‐T100) for 5 min on ice in PBS. After washing out viability dye. Cell cycle analyses were performed using a Click‐iT Plus EdU Alexa Fluor 647 Flow Cytometry Assay Kit (C10635; Invitrogen). Data were acquired on a BD Fortessa flow cytometer and analyzed with FlowJo 10.8.1 software.

### 4.5 Lipid Raft Staining

Lipid raft staining was performed using a Vybrant Alexa Fluor 555 Lipid Raft Labeling Kit (V34404; Invitrogen). Cells were analyzed by FACS.

### 4.6 Statistical Analysis

All statistical analyses were performed with GraphPad Prism software V10. To compare two groups, *p* values were determined using Student's *t*‐tests (two tailed). To compare more than two groups, one‐way analysis of variance (ANOVA) followed by Tukey's test was applied. For comparisons involving two factors, a two‐way ANOVA was performed, followed by Dunnett's multiple comparisons test. Differences between groups were considered significant for *p* values < 0.05.

## Author Contributions

**Zhenhan Zhu:** data curation (equal), formal analysis (equal), investigation (equal), writing – original draft (equal). **Wenxia Jiang:** data curation (equal), investigation (equal), methodology (lead), writing – review and editing (equal). **Jiehao Zhou:** investigation (equal), writing – review and editing (equal). **Alexander Robert Maldeney:** data curation (equal), writing – review and editing (equal). **Jingru Liang:** formal analysis (equal). **Jing Yang:** methodology (equal). **Wei Luo:** conceptualization (lead), investigation (lead), project administration (lead), supervision (lead), writing – original draft (lead).

## Conflicts of Interest

The authors declare no conflicts of interest.

## Data Availability

All data generated or analyzed during this study are included in this manuscript and figure files.
